# The appropriateness of the treatment setting for the inpatient post-acute treatment of alcohol dependence disorders in Switzerland

**DOI:** 10.1186/1752-4458-3-16

**Published:** 2009-07-01

**Authors:** Astrid Rossegger, Anne Keller, Michael Odenwald, Jérôme Endrass

**Affiliations:** 1 Psychiatric/Psychological Service, Criminal Justice System Canton of Zurich, CH-8090 Zurich; 2 Forel-Hospital, Islikonerstr. 5, CH-8548 Ellikon; 3 University of Constance, 78467 Constance, Germany

## Abstract

**Background:**

In Switzerland, a total of 1'000 patients a year are treated for alcohol-dependence in specialized institutions. Though the current literature suggests favoring outpatient treatment, whether outpatient or inpatient treatment is more efficient cannot be answered generally. For Germany, "AWMF"-treatment guidelines were formulated in order to treat patients with substance use disorders in the appropriate treatment settings. The aim of the present study was to test the hypothesis that the majority of patients treated in the largest specialized institution for alcohol abuse treatment in Switzerland were treated in the appropriate setting.

**Methods:**

All completed treatments conducted in the Forel-Hospital – the largest clinic of its kind in Switzerland – between the 1st of January 2004 and the 20th of December 2006 were included in the investigation (n = 915). Patient and treatment characteristics were gathered using the information from the PSYREC and act-info questionnaire. The AWMF criteria were operationalized on the basis of the questionnaire.

**Results:**

Applying the AWMF criteria resulted in the emergence of three groups: 73.7% of the study sample could clearly be assigned to the inpatient treatment group, and for 7.5% there was evidence supporting the allocation to an outpatient treatment setting. In 18.8% of the cases, however, the AWMF criteria did not allow an assignment to either of the treatment settings. Of the total sample, 18.5% of all patients apparently did not profit from the inpatient treatment setting, whereas for the vast majority (81.5%), a therapeutic progress was documented. In those patients who, according to the AWMF guidelines, did not need an inpatient setting, a larger proportion improved than in the group of the patients who needed an inpatient treatment in a specialized hospital. Furthermore, the logistic regression analyses revealed that the less severe the clinical state of a patient upon admittance, the higher the odds of improvement during the hospital stay.

**Conclusion:**

The results serve as evidence that for at least three out of four patients treated in the investigated specialized institution, an inpatient treatment was appropriate. The principal reason for the necessity of an inpatient treatment setting was that this hospital population showed severe psychiatric, somatic or social irregularities. Only a very limited number of patients hospitalized in a specialized institution for the treatment of alcohol-related disorders can be treated in an outpatient setting.

## Background

The treatment of alcohol-related disorders can be divided into acute and post-acute treatment. An acute treatment consists of interventions designed to reduce or eliminate specific symptoms, most commonly withdrawal symptoms. Since alcohol-related disorders consist of somatic and psychiatric symptoms, acute care must not only consider issues of detoxification, but also psychotherapeutic treatment. In the German-speaking language area, the integration of different treatment elements in the acute phase is called "qualified withdrawal treatment" (qualifizierte Entzugsbehandlung) [1,2]. The principal goal of this treatment perspective is for the patient to develop insight into his or her illness and to therefore develop the motivation for treatment [2]. Most acute alcohol-related care is performed in an acute ward. Typically, rehabilitative measures or post-acute treatment are performed immediately after an acute intervention, though an acute intervention is not a prerequisite for post-acute treatment.

Post-acute treatment focuses on long-term stability and thus relapse prevention. Post-acute treatment uses interventions designed to limit physical as well as psychological impairment, and also focuses on improving the cognitive and motivational functioning level of chronic alcohol abusers [3]. The foremost treatment goal is social reintegration, in the sense that the individual would be capable – to a certain degree – of participating in the job market and would also be able to organize his or her household without depending on professional help. In order to facilitate sustainable treatment success, aftercare programs supplement post-acute treatment [4]. Whether post-acute patients are better treated in an inpatient or outpatient setting has been a frequent topic of debate among researchers in this field [5-8].

The majority of published articles in the field suggest favoring outpatient treatment [9]. A quite recent Swiss multicenter study showed that of 915 patients treated in an inpatient setting, only 25% showed an improvement after seven years of follow-up [10]. Different follow-up studies, investigating the treatment effect of outpatient units, showed a relatively low figure of relapse: 49% after 24 months in the study by Soyka [11] and 53% after 18 months in the investigation by Mundle [12]. For Germany, it could be shown that patients treated in an outpatient setting were more likely to live in stable living conditions and less likely to be unemployed. However, these patients consumed more alcohol than patients treated in an inpatient facility and were less frequently treated prior to admission [13].

From an economic perspective, an outpatient treatment is preferable if there is no evidence that an inpatient treatment could lead to a better outcome. There is, however, a consensus that patients with severe psychiatric disorders and/or a lack of social support have a better outcome in inpatient treatment, whereas patients with a reduced severity of psychiatric symptoms and/or a high degree of social support would profit equally from an outpatient treatment [14]. There is also empirical evidence, that combinations of different treatment settings are best suited to achieve sustainable therapeutic effects [15]. Accordingly, objective and validated guidelines were developed in order to place patients with substance use disorders in appropriate treatment settings.

One of the comprehensive systems developed to improve the matching of substance abuse patients to various levels of care is the American Society of Addiction Medicine (ASAM) Patient Placement Criteria [16]. The ASAM placement system provides guidelines for assessing patients in two medical problem areas (acute intoxication/withdrawal and physical complications) and in four psychosocial problem areas (emotional/psychiatric complications, treatment acceptance, relapse potential, and recovery environment) [17]. In Germany, treatment guidelines were formulated by the Association of the Scientific Medical Societies (AWMF). AWMF is an association made up of 152 scientific societies from all areas of medicine. Through the separate scientific medical associations, AWMF has been coordinating the development of medical guidelines of diagnostics and therapy since 1995. AWMF is the national member for Germany in the Council for International Organizations of Medical Sciences (CIOMS) at the WHO in Geneva.

The treatment guidelines regarding the post-acute treatment of alcohol dependence are based on the respective American Psychological Association (APA) criteria for post-acute treatment [18]. According to the AWMF-criteria, there are several risk factors that, if present, qualify an individual for inpatient treatment: 1) Severe somatic, psychiatric or social disorder, 2) lacking social support, 3) no occupational integration, 4) unstable housing conditions, and 5) repeated relapses during outpatient treatment. An outpatient treatment setting is recommended if the following criteria are met: 1) The social environment offers an adequate support (e.g. stable living conditions), 2) there are no destructive or pathogenic influences in the social environment, 3) the patient is capable of participating in the treatment and is compliant with the treatment plan (by upholding sobriety), and 4) the ambulant treatment setting is explicitly preferred by the patient.

The guidelines are based on a systematic review of the literature, expert judgment and ultimate consensual agreement. So far, the usefulness of these guidelines has not yet been empirically investigated – at least not in the German-speaking language area.

In Switzerland, 20% of the adult population has developed high-risk alcohol consumption behavior. As a result of excessive alcohol consumption 13'634 predominantly inpatient treatments were carried out in Swiss hospitals in 2005. A total of 1'000 patients a year are treated in specialized institutions for alcohol-dependence [19,20].

The aim of the present study was to test the hypothesis that the majority of patients treated in the largest specialized institution for alcohol abuse treatment in Switzerland were treated in the appropriate setting according to the AWMF criteria. The alternative hypothesis was that these patients were not treated in the appropriate setting and could therefore just as well have been treated in an outpatient setting. The hypothesis was formulated as a directional hypothesis, in the sense that it was assumed that no patient still needing acute medical attention was accepted into the treatment program. Therefore, it was only investigated whether there were patients for whom an outpatient setting would have sufficed.

## Method

### Study sample

The data base (infodrog 2009) of the Swiss "Federal Office of Public Health" lists 21 institutions in Switzerland, which offer inpatient alcohol detoxification or treatment. Of these institutions, 4 are general psychiatric hospitals offering alcohol treatment without running a specialized addiction unit. The remaining 17 institutions can be split into specialized hospitals (n = 8) and specialized wards within psychiatric hospitals (N = 9). 13 of these institutions can only treat a small number of patients simultaneously (n<20). One out of four provide an alcohol specific treatment setting (n = 5). 10 facilities offer post-acute treatment. In summary, there are only two specialized institutions in Switzerland that a) offer an alcohol-specific treatment setting, b) provide a post-acute treatment plan and c) offer more than 20 treatment places at a time. The larger of these two institutions is the Forel-Hospital with 93 beds and is the only hospital of its kind in the northern part of Switzerland.

In the Forel-Hospital (named after a pioneer of Swiss psychiatry, Auguste Forel), patients with an alcohol dependence syndrome are treated in an inpatient setting. The Forel-Hospital is the largest clinic of its kind in Switzerland, performing one out of four inpatient treatments in this particular setting. Patients with schizophrenic disorders, lacking capacity to follow a conversation in German, severe cognitive impairment, need of nursing care, acute risk of suicide or violence towards others do not qualify for an inpatient treatment at the Forel-Hospital.

All treatments of patients discharged from the Forel-Hospital between the 1st of January 2004 and the 20th of December 2006 were included in the investigation.

In the study period, a total of 995 treatments were initiated in the Forel-Hospital, of which 80 were not completed by the end of the study period. This led to a final dataset of 915 completed treatments comprising 858 patients.

### Data collection

Patient and treatment characteristics were gathered using the information from the Psychiatric Patient Record (PSYREC) [21] and Addiction Care and Therapy Information (act-info) [20] questionnaire. Both documentation systems are the standard instruments in the Canton of Zurich (PSYREC) and Switzerland (act-info) for monitoring the psychiatric treatment and specialized treatment of dependence disorders.

PSYREC is the central psychiatric register that started in 1974 and covers all mental health services in the Canton of Zurich/Switzerland, a catchment area of about 1.2 million people. All psychiatric hospitals in the Canton of Zurich are legally mandated to report admissions and discharges to the register. PSYREC assesses information on the treatment, the treatment outcome, and basic socio-demographic information of all patients treated in a psychiatric facility in the Canton of Zurich. This data is transferred to the Swiss Federal Department of Statistics and is pooled with the data from other regions in Switzerland. In order to be eligible for pooling, the PSYREC items are thus adapted to the items that were assessed in the Swiss National Psychiatric Census.

Act-info is a standardized national monitoring system for outpatient and inpatient addiction care and was established in 2004. The documentation covers the situation of patients prior to initiation and on completion of treatment or counseling. Act-info is financed by the Swiss Federal Office of Public Health and was developed using the following questionnaires: a) the Treatment Demand Indicator Standard Protocol (TDI) from the European Monitoring Centre for Drugs and Drug Addiction [22], b) the Documentation Standards III for the evaluation of addictive treatment [23] from the German Society for Addiction Research and Addiction Therapy [23], c) the Addiction Severity Index [24], d) former questionnaires of the partial statistics SMBAD, SAKRAM/CRISA, FOS, Heroin-assisted treatment (HAT) and the national methadone statistics, e) selected, standardized screening tests, such as the Alcohol Use Disorders Identification Test (AUDIT) [25] or the Fagerstrom Test [26].

Whereas the PSYREC is scored solely by the therapist, the act-info documentation system includes a small self-report questionnaire. Both instruments use ICD-10 diagnosis and were administered at the time of admission and discharge. The analysis of the data – including the operationalization of the AWMF criteria – is based solely on the information obtained by these two instruments.

### Operationalization of the AWMF criteria for inpatient post-acute treatment

The AWMF guideline lists five criteria that, if met, qualify an addicted person for an inpatient post-acute treatment [3]. These criteria are not contained in the act-info and PSYREC documentation systems. In order to control whether the inclusion criteria for post-acute treatment in the Canton of Zurich meet the AWMF criteria, the variables were operationalized as follows:

Criterion "inpatient 1" requires the presence of a severe somatic, psychiatric or social disorder. A severe somatic disorder was scored if the patient suffered from a potentially life threatening disease (liver cirrhosis, esophageal varices, pancreatitis, cardiomyopathy) or from a very severe level of dependence at the time of admission – compared to other patients with that disorder. A severe psychiatric disorder was scored if the patient also suffered from an acute psychiatric disorder which had emerged at least 30 days prior to admission (major depression, anxiety disorder, suicidal ideations and attempts). Severe social problems were scored if patients had lived alone for at least six months *and *had been unemployed for a period of at least 30 days prior to admission.

Criterion "inpatient 2" requires a lack of social support and was scored if the patients stated, either at the time of admission or at the time of discharge, that they did have nobody they could turn to in case of an emergency.

Criterion "inpatient 3" addresses the absence of occupational integration and was scored if patients were unemployed in the last month prior to admission or were laid-off during their stay at the Forel-Hospital. Since Swiss law requires a waiting period of at least six months before an employee on sick leave can be discharged, those patients who lost their employment during their stay at the clinic were de facto unemployed several months prior to admission.

Criterion "inpatient 4" tackles unstable housing conditions. It was scored if patients had been institutionalized during the six months prior to admission or had lost their apartment and been homeless at the time of admission. Furthermore, if a patient had been living in such unstable conditions and was then evicted during his or her stay at the Forel-Hospital, it was also scored as "unstable living conditions".

Criterion "inpatient 5" requires repeated relapses during outpatient treatment and was scored if at least two previous post-acute treatments or at least two prior detoxification programs had failed.

### Operationalization of the AWMF criteria for outpatient post-acute treatment

The AWMF guideline lists four criteria that, if met, qualify an addicted person for an outpatient post-acute treatment [3]. These criteria are not contained in the act-info and PSYREC documentation systems. On the basis of the available data it was not possible to score two of the AWMF guideline criteria for outpatient treatment (criterion 3 and 4). The criteria 1 and 2 were operationalized as follows:

Criterion 1 requires an adequate support from the social environment (such as stable living conditions). It was assumed that this criterion was met, if a patient had lived with family members or significant others six months prior to admission and would be able to return to the same living conditions at the time of discharge.

Criterion 2 requires the absence of destructive or pathogenic influences in the social environment. This was assumed to be present, if a patient was 1) living in a stable relationship or living with a family member *and *2) was satisfied or very satisfied with his or her circle of friends and acquaintances.

### Operationalization of the treatment outcome

Upon admission to the Forel-Hospital each patient is routinely screened with respect to the severity of the disorder. At discharge, the therapists are required to document the treatment progress. The information regarding the treatment outcome was taken from the PSYREC. At the time of entrance into the clinic, the severity of the patient's substance dependence in comparison to other patients is assessed by a member of the treatment team using the PSYREC. At the time of release from the clinic, a member of the treatment team assesses whether the severity of the substance dependence has improved.

Both questions in the PSYREC are taken from the "Clinical Global Impressions" CGI rating scale [27].

If patients show at least a "moderate improvement" (as assessed by the PSYREC rating scale) in their health status compared to the status at admission, it is scored by the treating psychotherapist as improvement. "No improvement" or "worse than at time of admittance" is scored as no improvement. According to the follow-up study carried out 9 years ago in the Forel-Hospital, the psychotherapist's assessment correlated with the effective outcome assessed 30 months later [28] and can thus be viewed as a proxy measure for treatment outcome.

### Statistical Analysis

All descriptive analyses and the inferential statistics (Chi-Square and logistic regression analyses) were performed with STATA SE 10.0.

## Results

### Socio-demographic characteristics

Roughly two thirds of the treatments were conducted with male patients (67.8%, n = 620 vs. n = 295 treatments of female patients). The mean age was 46 years (SD: 9.3, range: 23 to 71 years). There was no significant gender-related difference with respect to the mean age (45.9 years for men and 46.7 years for women). At the time of admission, 29.8% (n = 271) of the patients were married, 34.6% (n = 315) were separated or divorced, and 33.2% (n = 302) were single. There were more single male than single female patients (38.3% vs. 22.5%). On the one hand, female patients tended to be divorced more frequently than men (41.8% vs. 31.1%). On the other hand, the prevalence of being married did not differ between the genders.

### Treatment history

82% (n = 750) of the patients treated in the Forel Hospital had a history of treatment for alcohol-related disorders prior to admission. 53% (n = 481) had received treatment in an outpatient setting, 38% (n = 344) in an inpatient setting and 64% (n = 584) had a history of detoxification. Before they were admitted to the current treatment in the Forel Hospital, 79% (n = 722) of the patients had undergone detoxification. The majority of these patients (n = 608, 84% of the detoxified patients) with the aid of medical treatment, while a small minority reported having undergone detoxification without the support of any drugs. Roughly half of the detoxifications (54%, n = 388) were performed in the acute ward of a psychiatric hospital, one fourth (126%, n = 190) took place in a general hospital, 8% (n = 59) in a specialized detoxification unit, and the rest in an outpatient setting. The vast majority of the patients treated in the Forel-Hospital (n = 72%, 518) were referred directly from the institution that was responsible for conducting the detoxification.

### Drinking behavior

At admission, 79.6% (n = 634) declared consuming alcohol at least four times per week. 67.2% (n = 523) reported a minimal daily consumption of six standard drinks, whereas one fourth (26.9%, n = 209) stated drinking six and more standard drinks only once a month, and 5.9% (n = 46) stated consuming six and more drinks per day less than once a month. 77.8% (n = 600) reported having suffered from a substantial loss of general performance in the twelve months before admission as a consequence of alcohol consumption. For the same time period, 66.3% (n = 516) declared consuming alcohol in the morning, and in 65.8% (n = 513) of the patients, the alcohol consumption has led to at least occasional amnesia.

### Treatment characteristics

The mean inpatient treatment duration was 104 days. Women stayed for shorter periods than men (95 vs. 108 days). 74.6% (n = 683) of the patients completed the regular clinic treatment program. At the time of discharge, 79.9% (n = 761) showed an improvement in their health status compared to the status at admission according to the treating psychotherapist. For 14.1% (n = 129), no improvement was documented and in 4% (n = 37) the health status even deteriorated. With respect to the improvement of the health status, there was no evidence of a gender effect.

### AWMF criteria for inpatient post-acute treatment

#### Criterion 1

A severe somatic, psychiatric or social disorder was found in 47.9% (n = 438) of the patients. There was no gender difference regarding this item (female: 50.2%, n = 148 vs. male: 46.8%, n = 290).

In detail: For 22.5% (n = 206) of the study sample, a potentially life threatening disease (liver cirrhosis, esophageal varices, pancreatitis, cardiomyopathy) or a very severe level of dependence at the time of admission was documented. Acute psychiatric disorders (major depression, anxiety disorder, suicidal ideations and attempts), which had emerged at least 30 days prior to admission, could be found in 17.1% (n = 156) of the cases. Severe social problems were found in 24.3% (n = 222) of the study sample.

#### Criterion 2

In 7.5% (n = 69) of the patients, potential social support was weak to non-existent. Male patients did not differ from female patients in regard to this criterion, 8.6% (n = 53) vs. 5.4% (n = 16).

#### Criterion 3

19.2% (n = 174) of the patients were unemployed in the last month prior to admission. A significantly larger proportion of men, i.e. 21.5% (n = 133) of the male patients – compared to 13.9% (n = 41) of the female patients – were seeking employment.

#### Criterion 4

In 12.0% (n = 110) of the study sample, the housing situation was unstable. There was no gender effect with respect to this criterion (male: 12.6%, n = 78; female: 10.8%, n = 32).

#### Criterion 5

50.1% (n = 458) of the patients had relapsed more than once during or after previous treatments. There was no gender specific influence with respect to this criterion (male: 49.2%, n = 305; female: 51.9%, n = 153).

Table 1 lists the proportions of the patients who fulfilled the aforementioned criteria. 24.4% (n = 223) of all the patients discharged from the Forel-Hospital between January 2004 and December 2006 did not meet any of the AWMF criteria for inpatient post-acute treatment, whereas 75.6% (n = 692) did. Roughly one third of the patients (31.9%, n = 292) fulfilled only one of the five criteria. 43.7% of the study sample (n = 400) fulfilled two or more criteria. Table 2 lists the sum of fulfilled criteria.

**Table 1:** Gender stratified proportions of patients who meet the respective AWMF criterion for inpatient treatment

Criterion		All (n = 915)		Male patients (n = 620)		Female patients (n = 295)	
		n	%	n	%	n	%

1	Somatic, psychiatric, and social impairment	438	47.9	290	46.8	148	50.2

2	Lacking social support	69	7.5	53	8.6	16	5.4

3	No occupational integration	174	19.2	133	21.5	41	13.9

4	Unstable living conditions	110	12.0	78	12.6	32	10.8

5	Recurrent relapses	458	50.1	305	49.2	153	51.9

**Table 2:** Severity of need for inpatient treatment according the AWMF criteria

Sum of fulfilled criteria	All		Male patients (n = 620)		Female patients (n = 295)	
	N	%	n	%	n	%

0	223	24.37	152	24.52	71	24.07

1	292	31.91	197	31.77	95	32.2

2	270	29.51	173	27.9	97	32.88

3	104	11.37	77	12.42	27	9.15

4	25	2.73	20	3.23	5	1.69

5	1	0.11	1	0.16	0	0

Total	915	100	620	100	295	100

It is evident that the mere presence of certain AWMF criteria regarding post-acute inpatient treatment makes an inpatient treatment necessary: If a patient suffers from a potentially life threatening disease or was evicted, the treatment options are limited and the room for maneuver is small. These criteria (criterion 1: "Somatic, psychiatric, and social impairment", criterion 2: "Lacking social support", criterion 4: "Unstable living conditions", criterion 5: "Recurrent relapses") can be referred to as the "conservative criteria" of the AWMF. On the other side, the sole presence of lacking occupational integration does probably not suffice to automatically require an inpatient treatment (criterion 3). For such cases, a day hospital could meet the therapeutic demands. Following this reasoning and narrowing the inclusion criteria for inpatient treatment, 73.7% (n = 674) of all patients would qualify for inpatient treatment, while 26.3% (n = 241) would not.

### AWMF criteria for outpatient post-acute treatment

In the group of the 223 patients who did not meet one of the conservative criteria for inpatient post-acute treatment, 43.5% (n = 97) met the first criterion for outpatient post-acute treatment (adequate support from the social environment) and 35.0% (n = 80) met the second criterion (absence of destructive or pathogenic influences in the social environment). In total, 26.9% (n = 60) of those patients, who did not need inpatient care, or 6.6% (n = 60) of the total sample, fulfilled both requirements for outpatient treatment. Gender did not have an effect on meeting the inclusion criteria for the outpatient treatment setting (27.0%, n = 41, of the male sub-sample vs. 26.8%, n = 19, of the female sub-sample). Table 3 lists the sum of fulfilled criteria for outpatient treatment.

**Table 3:** Gender stratified proportions of patients who meet the respective AWMF criterion for outpatient treatment

Criterion		All (n = 223)		Male patients (n = 152)		Female patients (n = 71)	
		N	%	n	%	n	%

1	Adequate support from the social environment	97	43.5	64	42.1	33	46.5

2	Absence of destructive or pathogenic influences in the social environment	80	35.9	58	38.2	22	31.0

Applying the AWMF criteria resulted in the emergence of three groups: 75.6% (n = 692) of the study sample could clearly be assigned to the inpatient treatment group and for 6.6% (n = 60) there was evidence which supported the allocation to an outpatient treatment setting. In 17.8% (n = 163) of the cases, however, the AWMF criteria did not allow the assignment to either of the treatment settings.

### Treatment outcome

Of the total sample, 18.5% of all the patients did apparently not profit from treatment at the Forel-Hospital, whereas for the vast majority (81.5%), a therapeutic progress was documented. In those patients who, according to the AWMF guidelines, did not need an inpatient setting, a larger proportion improved than in the group of the patients who needed an inpatient treatment in a specialized hospital. Table 4 shows the treatment outcome in connection with the AWMF classification.

**Table 4:** Treatment outcome and AWMF classification

Treatment outcome			Criteria fulfilled according to AWMF guidelines			
			
			Inpatient criteria		Outpatient criteria	
	N	%	N	%	N	%

No improvement	166	18.5	146	21.5	4	6.7

Improvement	731	81.5	533	78.5	56	93.3

Total	897	100	679	100	60	100

A series of bivariate logistic regression analyses revealed that the odds of therapeutic progress in the Forel-Hospital increased when the patients did not necessarily need an inpatient setting. More specifically, the odds for a successful treatment outcome was reduced for those patients showing a severe somatic, psychiatric, and social impairment, as well as for those patients living in unstable living conditions and for patients who had recurrent relapses. On the other hand, patients without pathogenic influences among friends and family had a higher chance of profiting from the hospital stay. Table 5 contains the results of the bivariate logistic regression analyses.

**Table 5:** Bivariate logistic regression analyses

		OR	p	95% CI	
Indication for inpatient treatment		0.37	0.00	0.22	0.60

Indication for outpatient treatment		2.46	0.00	1.44	4.18

Single criteria for inpatient treatment setting	Somatic, psychiatric, and social impairment	0.56	0.00	0.40	0.79
	
	Lacking social support	0.69	0.22	0.38	1.24
	
	No occupational integration	0.86	0.49	0.57	1.31
	
	Unstable living conditions	0.36	0.00	0.23	0.55
	
	Recurrent relapses	0.57	0.00	0.40	0.80

Single criteria for outpatient treatment setting	Adequate support from the social environment	1.39	0.09	0.95	2.03
	
	Absence of pathogenic influences in the social environment	2.01	0.00	1.30	3.12

## Discussion

Alcohol dependence is a chronic recurrent disease. If patients suffer from alcohol-related somatic disorders, the treatment takes place in regular (not specialized) hospitals for acute care. Very few patients are treated in a specialized institution and can thus profit from specialized interventions [29]. Whether outpatient or inpatient post-acute treatment is more efficient cannot be answered generally, though the current literature suggests favoring outpatient treatment [9]. There is a consensus that there is a variety of treatment methods and settings and that the ideal treatment has to be defined for each patient group. For some patients, an outpatient treatment can suffice, whereas for other patients a hospital stay is necessary. The AWMF developed guidelines which can help clinicians to decide which post-acute treatment setting is the most appropriate.

In the present study, we tested the hypothesis that patients, who were treated in specialized institutions for alcohol abuse, were treated in the appropriate setting. In order to test the hypothesis, the treatments were classified using the AWMF criteria for post-acute treatment. According to the AWMF, there are several risk factors that, if present, qualify for inpatient treatment: 1) Severe somatic, psychiatric or social disorder, 2) lacking social support, 3) no occupational integration, 4) unstable housing conditions, and 5) repeated relapses during outpatient treatment.

Three out of four investigated inpatients fulfilled at least one of the five inclusion criteria. Applying a more conservative approach than the AWMF required, and thus leaving out the lacking occupational integration, the result remained robust. Again, three out of four inpatients were identified as a population needing a post-acute inpatient setting. Accordingly, for roughly one fourth of the investigated inpatients, the necessity of a treatment in a post-acute hospital could not be identified. Since these patients did not match international criteria for inpatient treatment, the question arises whether these patients are best treated in an outpatient setting. Hence, all the patients who did not meet the criteria for inpatient treatment were investigated with the intention of assessing whether they could just as well have been treated in an outpatient setting.

To investigate this, the AWMF guidelines for outpatient treatment were used. Following the AWMF guidelines, an exclusive outpatient treatment can be recommended if four criteria are met: 1) The social environment offers an adequate support (e.g. stable living conditions), 2) there are no destructive or pathogenic influences in the social environment, 3) the patient is capable of participating in the treatment and is compliant with the treatment plan (by upholding sobriety), and 4) the ambulant treatment setting is explicitly preferred by the patient. In the context of the present investigation, only the first two inclusion criteria could be investigated.

So far, it has not been possible to get a precise estimate of the proportion of patients treated in an inpatient facility, who would have benefitted just as well from an outpatient treatment setting. For now, it is reasonable to assume that, at best, an outpatient setting would have sufficed for one out of four patients. When interpreting the results of this investigation, it is important to bear in mind that the inclusion criteria for inpatient treatment are "either – or" criteria, whereas all four outpatient treatment criteria have to be fulfilled in order to qualify for outpatient treatment. 6.6% of the investigated sample fulfilled at least two out of the four inclusion criteria. Whether those patients would have also met the remaining two criteria cannot be answered at this point. It is thus necessary in future research to develop a questionnaire, which allows the assessment of all four outpatient treatment criteria.

Aside from the above-mentioned methodological limitations, these results serve as evidence that an inpatient treatment was appropriate for at least three out of four patients treated in the investigated specialized institution.

Even though the empirical basis for outpatient treatment is sound, only a limited number of patients hospitalized in a specialized institution for the treatment of alcohol-related disorders can be treated in an outpatient setting. Also, it is noteworthy that 90% of those patients in our study, who fulfilled the two AWMF criteria for outpatient treatment improved during inpatient treatment. Furthermore, logistic regression analyses revealed that the less severe the clinical state of a patient was upon admittance, the higher the odds of improving during the hospital stay. This result thus suggests that inpatient treatment, as performed in a specialized institution, does not increase the dependence of the patients. It could have been assumed that rather long hospital stays increase the dependence of patients, hinder them in developing sustainable coping strategies and may in some cases even lead to mild forms of hospitalism. Why does the interpretation of the findings suggest that the healthier members of the hospital population profit from the hospital stay? The answer is probably multifaceted. Firstly, the degree of control in the hospital is very loose. The hospital is located in a village, the wards and the rooms are not locked, and there is only one hospital employee who is present at night for emergencies. Hence, patients who are not adequately motivated may relapse more easily. Secondly, the requirements for the psychotherapeutic group and single sessions are rather high. Patients have to be able to express themselves, talk about their emotions and needs, and actively participate in the therapeutic assignments. And finally, an important part of the therapeutic program consists of various levels of exposure, such as spending the weekend at home and meeting friends and family. Being able to cope with adverse living conditions and thus avoiding negative emotions as well as cognitive distortions, is an important resource for relapse prevention [30]. Transferred to the problem of which is the appropriate treatment setting, it becomes evident that while an inpatient treatment setting may protect from adverse living conditions, the outpatient setting on the other hand, enables the training of necessary coping strategies which can help prevent relapses.

### Limitations

So far, the obtained results were derived from the population of one institution. Since this hospital is the only specialized institution serving a large geographical region (population size of 1.2 million) there is no evidence suggesting that the investigated sample was severely biased. However, replication studies investigating other hospital populations of specialized institutions such as the Forel-Hospital are needed.

A further potential limitation is the use of the AWMF criteria. Even though these criteria were carefully selected and empirical studies using similar criteria showed a certain degree of validity [16], studies addressing the issue of the validity regarding these criteria are necessary. Still, even when accounting for the limitations mentioned above, it can be assumed that only a limited proportion of patients treated in a post-acute inpatient setting can benefit from a post-acute outpatient treatment.

Finally, the study design did not permit a direct comparison with other studies investigating the outcome of inpatient and outpatient treatment settings [9-12].

## Conclusion

The results serve as evidence that for at least three out of four patients treated in the investigated specialized institution, an inpatient treatment was appropriate. The principal reason for the necessity of an inpatient treatment setting was that this hospital population showed severe psychiatric, somatic or social irregularities. Probably only a very small number of patients hospitalized in a specialized institution for the treatment of alcohol-related disorders could alternatively have been treated in an outpatient setting.

## Competing interests

The authors declare that they have no competing interests.

## Authors' contributions

AR has given substantial contributions to the conception and interpretation of the data and helped draft the manuscript and furthermore, analyzed the data. AK, BG and MO have been involved in revising the manuscript critically. JE was substantially involved in carrying out the study design, analyzing the data, interpreting the results and writing the manuscript. All authors have read and approved the final manuscript.

## References

[B1] Croissant B, Joas A, Kohler F, Raben R (2003). [Patient with alcohol problems. Ignoring the signs or addressing them?]. MMW Fortschr Med.

[B2] Mann K, Loeber S, Croissant B, Kiefer F (2006). Qualifizierte Entzugsbehandlung von Alkoholabhängigen: Ein Manual zur Pharmako- und Psychotherapie.

[B3] Mundle G, Banger M, Mugele B, Stetter F, Soyka M, Veltrup C (2003). AWMF-Behandlungsleitlinie: Aktubehandlung alkoholbezogener Störungen. Sucht.

[B4] Greyer D, Batra A, Beutel M, Funke W, Görlich P, Günthner A (2006). AWMF-Leitlinie: Postakutbehandlung alkoholbezogener Störungen. Sucht.

[B5] McLellan A, Luborsky L, Woody G, O'Brien C, Druley K (1983). Predicting response to alcohol and drug abuse treatments. Role of psychiatric severity. Arch Gen Psychiatry.

[B6] McLellan A, Woody G, Luborsky L, O'Brien C, Druley K (1983). Increased effectiveness of substance abuse treatment: A prospective study of patient-treatment "matching". Journal of Nervous and Mental Disease.

[B7] Miller W, Hester R (1986). Inpatient alcoholism treatment. Who benefits?. Am Psychol.

[B8] Nace E (1990). Inpatient treatment of alcoholism: a necessary part of the therapeutic armamentarium. Psychiatr Hosp.

[B9] Bottlender M, Soyka M (2005). Outpatient alcoholism treatment: predictors of outcome after 3 years. Drug Alcohol Depend.

[B10] Maffli E (1996). 7 Jahre nach der stationären Behandlung. Abhängigkeiten.

[B11] Soyka M (1999). Efficacy of outpatient alcoholism treatment. Addiction.

[B12] Mundle G, Brugel R, Urbaniak H, Langle G, Buchkremer G, Mann K (2001). [Short- and medium-term outcome of outpatient treatment of alcohol dependent patients. A 6-, 18- and 36-month follow-up]. Fortschr Neurol Psychiatr.

[B13] Welsch K, Sonntag D, Suchtfragen DHf (2004). Jahresstatistik der professionellen Suchtkrankenhilfe. Jahrbuch Sucht 04.

[B14] Pettinati H, Meyers K, Jensen J, Kaplan F, Evans B (1993). Inpatient vs outpatient treatment for substance dependence revisited. Psychiatr Q.

[B15] Mann K, Günthner A, Nissen G (1994). Neue Aspekte in der Behandlung Alkoholabhängiger. Abhängigkeit und Sucht.

[B16] Turner WM, Turner KH, Reif S, Gutowski WE, Gastfriend DR (1999). Feasibility of multidimensional substance abuse treatment matching: automating the ASAM Patient Placement Criteria. American Society of Addiction Medicine. Drug Alcohol Depend.

[B17] McKay J, Cacciola J, McLellan A, Alterman A, Wirtz P (1997). An initial evaluation of the psychosocial dimensions of the American Society of Addiction Medicine criteria for inpatient versus intensive outpatient substance abuse rehabilitation. J Stud Alcohol.

[B18] Schmidt LG, Gastpa M, Falkai P, Gaebel W, Eds (2006). Evidenzbasierte Suchtmedizin.

[B19] SFA, ISGF, ISPM act-info – Entwicklung der Altersstruktur der Klienten und Klientinnen des Schweizer Suchthilfesystems. http://www.actinfo2009.ch/textes/actinfo_Artikel%20spectra_081203.pdf.

[B20] sfa/ispa act-info Eintrittsfragebogen für den stationären Alkohol- und Medikamentenbereich (SAKRAM). http://www.sfa-ispa.ch/DocUpload/Eintrittsfragebogen_actinfo_Residalc1_2.pdf.

[B21] Psychiatriepatientenstatistik (PSYREC). http://www.pmh.unizh.ch/.

[B22] EMCDDA (2000). Treatment Demand Indicator Standard Protocol 2.0.

[B23] DG-Sucht (2001). Documentation Standards III for the evaluation of addictive treatment. Sucht.

[B24] McLellan AT, Kushner H, Metzger D, Peters R, Smith I, Grissom G, Pettinati H, Argeriou M (1992). The Fifth Edition of the Addiction Severity Index. J Subst Abuse Treat.

[B25] Babor T, de la Fuente J, Grant M (1992). AUDIT: The Alcohol Use Disorders Identification Tests: Guidelines for use in primary health care.

[B26] Heatherton TF, Kozlowski LT, Frecker RC, Fagerstrom KO (1991). The Fagerstrom Test for Nicotine Dependence: a revision of the Fagerstrom Tolerance Questionnaire. Br J Addict.

[B27] CIPS, Ed (1986). Internationale Skalen der Psychiatrie.

[B28] Christoffel U, Liechti U, Meyer T, Sieber M (1999). Kontrolliertes Trinken und Kontrollüberzeugungen. Bulletin der Forel Klinik.

[B29] Soyka M, Heinrich K (2008). Alkoholismus – Missbrauch und Abhängigkeit Entstehung – Folgen – Therapie.

[B30] Marlatt GA, George WH (1984). Relapse prevention: introduction and overview of the model. Br J Addict.

